# Editorial

**DOI:** 10.2174/157340311795677734

**Published:** 2011-02

**Authors:** Gert J. de Borst

Cardiovascular disease (CVD) represents the number one and leading cause of death globally, and the majority of CVD is caused by atherosclerosis. Atherosclerosis is a systemic disease that leads to myocardial infarction, stroke and lower limb ischemia. Pathological studies have given insight to development of atherosclerosis and the importance of local plaque vulnerability and cardiovascular events. Due to the burden of cardiovascular disease, identification of the individual patient at risk for cardiovascular events and treatment stratification is needed. Furthermore, efforts at primary prevention for major risk sources and secondary prevention of recurrence are very important. For example, following stroke due to carotid artery stenosis, the likelihood of recurrence is relatively high (up to 10% for early embolic recurrence) but currently we have no options to predict who will and who will not suffer a secondary event. Although large series of patients have been reported in literature, some clinical aspects of the natural course of the disease are still poorly defined, creating doubts how to select the patient that will benefit from revascularization considering perioperative morbidity from the procedure itself.

The predictive power of classical risk factors is limited, especially in patients with manifest atherosclerosis. Prediction models are often made by non patient treating authors; making them distant from what it is all about: daily care for the individual patient. Imaging modalities have focused on the characteristics of the vulnerable plaque, and options for non-invasive imaging need to be further explored. Serum biomarkers have also been studied extensively. In line with the important relation between vulnerable plaques and cardiovascular events, plaque biomarker studies have been initiated. These longitudinal studies are based on the concept, that a vulnerable plaque contains predictive information for future (cardiovascular) events. Results look promising and plaque markers can be used to subsequently develop imaging modalities to identify patients at relatively high risk. 

The objective of this supplement is to present an update on relevant aspects related to clinical manifestations, biological characteristics, implications of biobanking for individual patient risk identification, and imaging developments, in order to contribute to the better understanding of individual patient directed medical care.

The most relevant aspects are discussed in the following 5 chapters: 1) “Risk scores for chest pain patients at the emergency room”. Chest pain is a common reason for presentation to the emergency department (ED). The vast majority of patients with chest pain are due to causes other than Acute Coronary Syndrome. The developed HEART score is specifically designed to stratify all chest pain patients and proved to be a strong predictor of event free survival on one hand and potentially life threatening cardiac events on the other hand.  2) Biomarkers derived from histological controlled studies have long time been skye-high…but there seem to be pitfalls, creating some disbalance. We hope you will find out reading the contribution from our Experimental Cardiology Department entitled “Plaque markers predictive for outcome: challenging the definition of the vulnerable plaque?; 3) With increasing life expectancy clinicians are more often confronted with patients of higher age. Octogenarians were often excluded from randomized trials comparing CAS to CEA because they were considered high-risk for revascularization. Conflicting results on the peri-procedural outcome of carotid revascularization in these patients have been reported, and Reichmann *et al*. summarize and evaluate whether age above 80 years should be an upper limit for indicating carotid revascularization in the manuscript “Octogenarians and carotid plaque: histologically based risk for adverse perioperative outcome?; 4) Future direction for adequate patient selection will be imaging guided treatment checked by the gold standard of histology. This approach will have a high impact for patients, health care systems and society. In patients with carotid artery stenosis histological plaque composition is associated with plaque stability and with presenting symptomatology. Preferentially, plaque vulnerability should be taken into account in pre-operative work-up of patients with severe carotid artery stenosis. However, currently no appropriate and conclusive (non-)invasive technique to differentiate between the high and low risk carotid artery plaque *in vivo *is available. and Den Hartog *et al*. propose a new study protocol entitled “The application of 7Tesla MRI in determining carotid plaque characteristics in patients with high grade carotid artery stenosis”; and 5) Biobanks are an extremely valuable resource that enables us to study the influence of both genetic and environmental factors on the development of multifactorial diseases such as atherosclerosis. The review by Scholtes *et al*. “Biobanking of vascular tissue: opportunities and pitfalls” will focus on the advantages and pitfalls in atherosclerotic biobanking.

Hopefully, all being interested in patient selection improvement and the role of biobanking working in the field of cardiovascular diseases will enjoy the contents of this mini-guest editorial and will find topics of personnal interest or food for thought and spirit for future investigations and research.

I would like to acknowledge in particularly Dr Jianyi Zhang, editor-in-Chief of the Current Cardiology Reviews series, for the kind invitation to participate in this editorial.

